# Stuck in a Time Warp? The Great Recession and the Socio-occupational Integration of Migrants in Spain

**DOI:** 10.1007/s12134-021-00914-1

**Published:** 2022-01-15

**Authors:** Juan Ramón Jiménez-García, Antonina Levatino

**Affiliations:** 1 Department of Political and Social Sciences, Universitat Pompeu Fabra & Migration Research Group, CIDOB, Barcelona, Spain; 2 Department of Sociology, Universitat Autònoma de Barcelona, Bellaterra, Spain

**Keywords:** Socio-occupational integration, Immigrant population, Labour market, The Great Recession, Spain

## Abstract

This article examines the socio-occupational integration of the immigrant population in Spain for a time span that, for the first time, includes the post-crisis period. Using the Spanish Labour Force Survey and conducting a socio-occupational analysis, we predict the probability that a migrant would be employed in one socio-occupational class over another in three periods: before, during and after the crisis. Our main research questions are as follows: (1) To what extent do migrants tend to be located in certain socio-occupational classes? (2) To what extent does the likelihood of belonging to a certain socio-occupational class differ according to immigrants’ places of origin? (3) Can differences be found in the likelihood of belonging to a certain socio-occupational class according to the places of origin before, during and after the Great Recession? The results show a very unequal distribution of immigrants in the socio-occupational structure according to their origin. While immigrants from Schengen Europe and North America are better located in the occupational structure, those from Eastern Europe and Africa are over-represented in the lower socio-occupational classes.

## Introduction

The consequences of the Great Recession on the Spanish labour market have aroused strong interest in the academic sphere, especially regarding its possible impact on the occupational and social situation of the most vulnerable groups. As some previous investigations have shown (see, e.g. Kogan, 2004), the immigrant population is very dependent on economic cycles and therefore may constitute one of the groups most affected by economic recessions. Given such evidence, it is not surprising that a large amount of research has focused on the impact of the recent economic crisis of 2008 on the employment situation of immigrants in Spain (Cebolla-Boado et al., 2015; Garrido et al., 2010; Muñoz-Comet, 2011). These investigations have shown that immigrants have generally been more at risk of losing their jobs than natives. However, the crisis has not affected all immigrants uniformly. Its effects seem to vary substantially according to nationality (Cebolla-Boado et al., 2015) and gender (Arranz et al., 2017; Muñoz-Comet, 2011). More recently, Jiménez-García (2018) found higher probabilities of access to the labour market by people with foreign nationality compared to natives during the crisis. The mechanisms behind this greater employability of immigrants compared to their native counterparts are not yet clear and defined. Most probably, the explanation is related to the type of jobs occupied by migrants, especially by women, who generally experience greater precariousness and fewer opportunities for job promotion (Bernardi & Garrido, 2008). The segmentation of the Spanish labour market would allow immigrants to maintain greater employability, particularly in precarious occupations in which there are no native workers (Martínez-Martín & Prior-Ruiz, 2011).

Although recent research has shown how the Great Recession has modified European labour markets, increasing job polarisation, and decreasing job opportunities and occupational mobility (Bisello et al., 2020; Fellini, 2017), to date we find no evidence of the existence of any study that has analysed the socio-occupational integration of immigrants in the Spanish labour market in a time span that includes the post-crisis period. This is unfortunate considering that (1) Spain has been one of the countries most heavily hit by the crisis in Europe and the OECD (Aristegui et al., 2017); (2) during the crisis, a number of neoliberal policies have been implemented in this country with the aim of modifying its labour market by cheapening labour costs and deregulating aspects such as dismissals (Muñoz-Comet & Martínez-Pastor, 2017; Pavolini et al., 2015); and (3) the implementation of austerity policies and the cuts of social rights have left many immigrants in situations of great vulnerability (Bruquetas-Callejo & Moreno, 2015; Pavolini et al., 2015).

This study addresses this gap by examining migrants’ socio-labour integration not only for the period preceding the crisis (2006 to 2008) or the period of the crisis (2010 to 2012), but also including the post-crisis period (2014 to 2016). To do this, we use microdata from the Spanish Labour Force Survey (INE, 2017a, b) and conduct, to our knowledge for the first time, a socio-occupational class analysis that aims to predict the probability of being located in one class over another. The research questions are as follows: (1) To what extent do migrants tend to be located in certain socio-occupational classes? (2) To what extent does the likelihood of belonging to a certain socio-occupational class differ according to immigrants’ places of origin? (3) Can differences be found in the likelihood of belonging to a certain socio-occupational class according to places of origin before, during and after the Great Recession?

The remainder of the paper is organised as follows. The “Theoretical Framework” section offers an overview of the theoretical framework, while the “Literature Review” section provides a literature review identifying gaps in existing research. In the “Methodology” section, the working hypotheses, the data and the analytical strategy are presented. The “Results” and “Discussion” sections display and discuss the results, respectively. The “Conclusion” section offers conclusions.

## Theoretical Framework

### Migrants’ Integration in Host Countries’ Labour Markets

Several studies have tried to explain migrants’ disadvantages in host countries’ labour markets with particular regard to the differences between natives’ and migrants’ wages, access to job opportunities and positioning in the socio-occupational structure.

A first approach derives from the neoclassical paradigm of human capital (Becker, 1962, 1964), according to which there are no barriers for social and labour mobility in the society and the labour market, and all individuals are allocated in the socio-occupational structure according to their human capital (Chiswick, 1978; Chiswick & Miller, 2002; Chiswick et al., 1997). The worse positioning of immigrants in the socio-labour structure is linked with the characteristics of their human capital and is due, for example, to the lower quality or highly context-sensitive education received in the origin country (Bernardi et al., 2011; Kanas & Van Tubergen, 2009), to immigrants’ weaker understanding of the host country labour market rules and style of practice (Chiswick et al., 2005; Vidal-Coso & Miret, 2014), to problems attached to the homologation and recognition of degrees which is often a slow, sometimes even impossible, process (Rinken et al., 2011) or to immigrants’ lack of language skills (Chiswick & Miller, 2002; Sanromà et al., 2008). According to Chiswick et al. (2005), immigrants from countries that are more similar to the destination country (because of historical and cultural links, because of the similarity of the education system and/or because of the common language) will experience this disadvantage less as their human capital is more easily spendable in the host country labour market. By investing time and effort in host societies, immigrants will acquire the skills, habits and abilities needed in order to become more suitable candidates for the best occupations (Dustmann & Fabbri, 2003). This way, their socio-economic status will gradually converge to that of natives. That is why this hypothesis is commonly referred to as the immigrant assimilation model^1^ (see, e.g. Chiswick et al., 2005).

The immigrant assimilation model has been challenged by the results of research which shows that, despite the time spent in the host country and the educational credentials, the immigrant population never reaches parity with the natives, either in the probability of accessing a quality job (Zorlu & Hartog, 2012; Sánchez-Domínguez & Fahlén, 2018) or in the possibility of reaching the same incomes (Okoampah, 2016). Furthermore, studies have shown that, even after taking into account immigrants’ educational qualifications and other relevant personal characteristics with respect to natives, an “ethnic or immigrant penalty” remains concerning employment access, wages or type of occupations held (Fellini, 2017; Heath & Ridge, 1983; Heath et al., 2000; Kogan, 2004). This can be due to factors that are difficult to analyse with survey data (e.g. how migrants are searching for work, their lack of relevant social capital, etc.) or to discrimination (Kogan, 2004).

Apart from these explanations, other theories more focused on labour market segmentation may also shed some light on the “ethnic penalty”. According to Bonacich (1972, 1976), ethnic antagonism emerges when different ethnic/racial groups whose hiring costs sensibly differ are competing for the same jobs. Employers’ preference to hire cheaper workers results in a highly segmented labour market where the most vulnerable workers, who are the cheapest ones, are restricted to specific occupations characterised by lower wages. As a consequence, the lowest-status jobs are normally occupied by the most vulnerable workers, i.e. members of ethnic and marginalised minorities, non-unionised workers, new immigrants, migrants from impoverished countries and undocumented migrants, whose work is priced lower. According to Doeringer and Piore (1971), migrants may be more ready than natives to accept jobs in the “secondary” labour market, i.e. jobs associated with lower wages, lower prestige or dangerous conditions. This could be the case because, for example, migrants may be seeking immediate monetary return and/or are less worried than natives about status in the host country (Kogan, 2004). While the initial approaches to segmented labour market theorised a dualistic model, more recent studies propose a theorisation in which the labour markets are divided into multiple layers (Rubery, 2007). It has been also argued that the fact that migrants can become associated with particular “segments” might contribute to the perpetuation of the labour market segmentation (Waldinger & Lichter, 2003). Recent research has reported differences between the jobs held by immigrants coming from Southern European countries and natives in the UK, Denmark and Germany, also highlighting wage inequalities (Felbo-Kolding et al., 2018). Research conducted in Belgium (Vertommen & Martens, 2006) highlighted differences between groups of different origins, arguing for an “ethno-stratified” labour market, divided into different ethnic layers, where people of different origins are more or less represented in better or worse professions. Other studies have found that migrants coming from Western countries are generally better integrated in the labour market than other migrants (Brodmann & Polavieja, 2010; Kogan, 2011).

### Approaches to Analyse the Labour Market Structure

As argued by Oesch (2003: 241), class schemas are among the most useful and “powerful” tools to analyse labour market structure and segments. Currently, two are the most used classifications, the Wright class schema and the one developed by Goldthorpe and colleagues. In the Wright class schema, inspired by the Marxist theoretical approach, classes are defined and categorized in terms of exploitation and domination. The Goldthorpe classification, inspired by the Weberian theoretical approach to class, is based on the analysis of social relations occurring in labour markets and productive units. This classification takes into account the characteristics of the employment, such as salary, educational level required, economic security attached to the type of employment, the individual’s position in the hierarchy of authority, the individual degree of autonomy at work and the size of the enterprise and the manual/non-manual nature of the work done (Domingo-Salvany et al., 2013). Because of its conceptual rationale and its pragmatic scope, the Goldthorpe class schema has become the most influential and widely used categorisation of class in European sociology and in international research of social classification (Oesch, 2003) and is also considered the reference framework for the study of social classes in Spain (e.g. Regidor, 2001).

## Literature Review

### Immigrants in the Spanish Labour Market

Spain has gone from being a migrant-sending country to a migrant-receiving country in a very short period of time (Freeman, 1995). Currently, of the almost 47 million inhabitants in Spain, 5 and a half million are foreigners, which in relative terms represent more than 11.4% of its population (INE, 2021). As in other Southern European countries, much of the population entry has been driven by the high demand for labour at specific moments (Arango, 2013), and it has been characterised by a high volume of undocumented migrants (Finotelli & Ponzo, 2018). The majority of these undocumented migrants regularise their status ex post through the possession of a labour contract (Finotelli & Arango, 2011). For this reason, migrants’ labour market inclusion is considered the most relevant factor for their integration in Southern European societies (Finotelli & Ponzo, 2018; Freeman, 2004).

In contrast with other European countries with a longer migration tradition, and similarly to other Southern European countries such as Italy, Spain is characterised by a highly segmented labour market characterised by a rapid insertion of migrants in the lowest-quality jobs (Ballarino & Panichella, 2015; Panichella, 2018). This results in a high occupational segregation and in a low occupational and social mobility (Ambrosini, 2001). Other structural features of the Spanish labour market contribute to this high occupational segregation: (1) the fragmentation of the productive sector into poorly protected micro-enterprises, where the control of irregularities and illegalities regarding hiring is minimal; (2) the existence of a very widespread underground economy that allows many undocumented workers to obtain employment; and (3) the extensive use of temporary contracts that allows employers to quickly adapt to changes in the economic cycle, in exchange for offering very little job stability to workers (García-Serrano and Malo, 2011).

In the recent decades, a large number of investigations have focused on the socio-labour integration of immigrants in the Spanish labour market. The first studies, traced back in the 1990s, highlight discrimination in the access to work and on working conditions of migrants compared to natives. For Cachón (1995), this discrimination comes from the legal framework itself, giving rise to an “institutional discrimination”. Later on, studies before the Great Recession placed Spain as a clear example of socio-labour integration (Amuedo-Dorantes & Rica, 2007; Izquierdo et al., 2009). Indeed, even though immigrants were concentrated in the most unstable and less qualified sectors (Bernardi & Garrido, 2008; Bernardi et al., 2011), their employment rates were similar to natives (Fernández and Ortega 2008). In this line, Pumares Fernández et al. (2006) highlighted that before the crisis, South Americans and immigrants from enriched countries were able to experience ascending social mobility processes after 5 years of residence. Similarly, Fernández and Ortega (2008) showed that upon arrival, immigrants had very high unemployment rates compared to Spaniards, but also showed that after 5 years since their arrival, they become similar. The authors however remarked that, regardless of the time spent in Spain, immigrants are more likely than natives to experience overeducation, a situation that occurs when worker owns an educational level that “exceeds that required for his or her occupation” (Capsada-Munsech, 2017: 2), and have greater chances of being employed in temporary jobs. Other studies (Parella, 2003; Sanromà et al., 2008; Solé & Parella, 2003) highlight that, regardless of residence time in Spain have worse wages than natives. They also emphasize that migrants constitute a heterogeneous group: while immigrants from rich countries enjoy working conditions similar to natives, immigrants from impoverished countries always occupy the lower labour segments, often experiencing racial discrimination (Parella, 2003).

Since the Great Recession of 2008, the focus has been placed on the impact of the economic crisis on the work trajectories and socio-occupational integration of migrants. In his descriptive analysis, Muñoz-Comet, (2011) shows how immigrants generally occupy the worst jobs in terms of wages and status. He also shows that immigrants suffer the most from economic recessions due to their younger age, their lower experience and their lower level of education. They are therefore more likely to lose their jobs during crises. Nonetheless, he also shows how immigrant women, especially those from Europe and Latin America, have much better resistance to such crises, when it comes to unemployment, than their male peers (Muñoz-Comet, 2011). This study confirms the findings of research conducted in a similar context, Italy, that has shown that immigrants had faced the Great Recession in a different way compared to natives (e.g., Bonifazi & Marini, 2014; Venturini & Villosio, 2018). In a similar vein, Cebolla-Boado et al. (2015) analysed the market value of the educational credentials held by migrants compared with native males for the period 2003 to 2012. They claim that migrants have been less able than natives to gain employment through their education since the Great Recession. In addition, they found important differences among migrants: East European migrants have much a greater probability of employment compared to Africans and Latin Americans. Similarly, Martínez-Martín and Prior-Ruiz (2011) found that immigrants from rich countries enjoy faster socio-labour integration than immigrants from impoverished countries who occupy the lowest job ranks and are hit harder by recessions.

As can be noted by this review, the topic has inspired a large amount of research. Nonetheless, existing studies have focused on the period before the crisis and the crisis years, but we did not find evidence concerning the Spanish case after the crisis. Furthermore, we find no study that has conducted an analysis that explores the probability of being located in the different socio-occupational classes.

### Migrants’ Employment After the Great Recession

To our knowledge, until now, no study has explored the conditions of migrants’ employment after the crisis for the Spanish case. However, if we look at other countries, we find some studies that have examined the condition of migrants’ employment during and after economic recessions. For the Swedish case, for example, Åslund and Rooth (2007) study how the condition of the economy when the migrant arrives affects economic integration in the long term, finding that those who enter a country during an economic downturn have a slower rate of economic and occupational advancement, which persists over decades. Venturini and Villosio (2018) analyse the characteristics of migrants’ employment before and after the Great Recession in Italy showing how the crisis exacerbated the high segmentation of the Italian labour market in terms of low-skilled, unstable, and poorly paid jobs. Fellini (2017) compares the insertion of immigrants in Italy and Spain in 2007 and 2012, finding that the risk of being unemployed or accessing medium and highly skilled non-manual jobs varies and that migrants from certain origins are more penalised than others. She also shows that, between these 2 years, in both countries, both natives and migrants experienced a decline in labour market outcomes and that, in Spain, the unemployment risk for migrants significantly increased.

Although it is not focused on migrants, the investigation conducted by Bisello et al. (2020) on employment and occupational transitions before, during and after the crisis (until 2013) in six European countries, including Spain, is also relevant for our research. The authors observe different patterns in the analysed countries. In the case of Spain, they observed how a great amount of mid-paid jobs were destroyed during the crisis, resulting in increased job polarisation afterwards; i.e. there was a decline of mid-paid jobs with respect to jobs at the top and bottom of the labour market structure (Fernández-Macías & Hurley, 2017).

## Methodology

### Research Questions and Working Hypotheses

Three research questions guide our research. The first two questions are as follows: To what extent do migrants tend to be located in certain socio-occupational classes? To what extent does the likelihood of belonging to a certain socio-occupational class differ according to immigrants’ areas of origin? Considering the theoretical framework, we expect that, (H1) even after controlling for educational level and work experience, compared to natives, migrants have a lower likelihood of belonging to higher socio-occupational classes and a higher likelihood of belonging to the lowest socio-occupational classes or of being unemployed (“ethnic penalty”). Moreover, we hypothesised that (H2) the likelihood of belonging to a certain socio-occupational class will differ according to the migrants’ origin, with some groups displaying a larger “ethnic penalty” than others (“ethnic-stratified labour market”).

The third research question concerns the comparison of before, during and after the Great Recession: Can differences be found in the likelihood of belonging to a certain socio-occupational class according to the areas of origin before, during and after the Great Recession? We expect (H3) an increased likelihood of unemployment for both natives and migrants during and after the crisis, as compared to the before-crisis period, and a decreased likelihood for both natives and migrants to belong to a middle socio-occupational class (“job polarisation”).

### Data

We use microdata coming from the Spanish Labour Force Survey (hereafter LFS) provided by the Spanish Statistical Office (INE, 2017a, b) for the period 2006 to 2016. These data are suitable for this investigation for different reasons. Firstly, the LFS survey format allows the analysis of not only declared employment, but also irregular employment. This work practice is very relevant when studying labour dynamics, especially of immigrants, who are often excluded from many data sources in the case of working without work permits. Secondly, thanks to the level of disaggregation of the occupations in the LFS, the socio-occupational class of the people surveyed can be studied very precisely. Moreover, contrary to other sources of data, in which immigrants are many times excluded, the LFS has a favourable proportionality that allows the situation of migrants in each socio-occupation to be studied in depth.

The LFS is a rotatory panel published quarterly and has a sample of 60,000 households (180,000 individuals approx.). Participants are selected through a two-stage sampling with stratification in the first-stage units for each of the 56 Spanish provinces. The first-stage units are the population census sections, while the second-stage units are the main family dwellings in which all the people residing in them are interviewed. In order to avoid attrition, each quarter, one-sixth of the people are renewed.^2^ Each household is interviewed up to a maximum of six quarters, i.e. a year and a half. Unfortunately, in the LFS, the identifiers (ID) of each household and each individual change every quarter. This may lead to repetitions of individuals in the sample. In order to avoid this common error, we selected only a quarter for each year under analysis. More specifically, we selected the second one because this quarter is the most stable in terms of employment and is not conditioned by seasonality.

### Methods and Variables

To predict migrants’ probabilities of belonging to a certain socio-occupational class before, during and after the Great Recession and test our hypotheses, we perform three multinomial logit regressions for three periods, obtained by merging the observations from different years: before the crisis (2006 and 2008), during the crisis (2010 and 2012) and after the crisis (2014 and 2016).

The dependent variable is *socio-occupational class*, which is a categorical variable that has been constructed from the professions described in the National Classification of Occupations^3^ (INE, 2011) disaggregated to three digits. The classification in seven socio-occupational classes is the one proposed by Domingo-Salvany et al. (2013) to adapt Erikson and Goldhorpe neo-Weberian occupational class structure (1992) to the Spanish case: I, higher grade university-level professionals and managers with 10 or more employees; II, lower grade university-level professionals and managers with less than 10 employees; III, intermediate professions, administrative employees and administrative management and other service support professionals; IV, small proprietors and self-employed workers; V, supervisors and workers in skilled technical occupations; VI, skilled and semi-skilled manual workers; and VII, unskilled manual workers. To these seven categories, we add a further one that includes people who are unemployed.

To explore differences between migrants’ groups, we used the variable *origin*. From the original variable contained in the LFS of “foreign nationality”, including more than 120 nationalities, we created six categories^4^: Spain, which groups people with Spanish nationality and those with dual nationality in which one of them is Spanish. Schengen Area, UK, Ireland and North America, which groups people coming from the 25 current Schengen member states (Spain is excluded), UK, Ireland, Canada and the USA; non-Schengen Europe, which groups people from non-Schengen European countries (except UK and Ireland); Africa; Central-South America, which includes people from the Caribbean, Central America and South America^5^; and Asia. We prefer to choose the variable “nationality” instead of “country of birth”, since people born abroad but with Spanish nationality enjoy the same legal and administrative status as people born in Spain.^6^

*Educational level* measures the highest level of studies reported by the respondents. It has three categories: low level, which includes illiterate people and people with primary education degrees; middle level, which comprises people with secondary education qualifications, professional training and certificates of professionalism; and high level, which includes people with academic degrees. The variable *work experience* is a continuous variable that refers to years of experience in the Spanish labour market. We prefer this variable over introducing a more generic variable on time spent in the host country, since this variable allows for a better comparison with natives and since we believe this constitutes the more accurate proxy of the extent to which an individual has accumulated relevant knowledge and experience, which is spendable in the labour market. It would be useful to control for whether the migrant received education or training in Spain or whether respondents changed occupational status in the Spanish labour market. This information, however, is not available. Similarly, it would be beneficial to be able to control for whether the respondent is working regularly or irregularly. Unfortunately, the database does not have any variable that allows us to distinguish it. All the models control for *gender*, as many studies seem to show that the socio-occupational integration of women may differ from that of men, especially during the crisis period.

The descriptive statistics of all the variables included in the analyses are provided in Table 1.

**Table 1:** Descriptive statistics

Categorical variables	Categories		Frequency	%
Socio-occupational class	I Higher grade professionals with more than 10 employees in their charge		34582	8.85
	II Lower grade professionals directors and managers of university level with less than 10 employees		43750	11.20
	III Routine non-manual employees, administrative employees and professionals of support to the administrative and other management services		79905	20.46
	IV Small proprietors and self-employed workers		31945	8.18
	V Lower-grade technicians, supervisors and workers in qualified technical occupations		44028	11.27
	VI Skilled, semi-skilled and manual workers		58065	15.57
	VII Semi-skilled and unskilled manual workers		233186	3.41
	Unemployed		82299	21.07
Educational level	Low level		58065	14.87
	Middle level		233186	59.70
	High level		99361	25.44
Origin	Europe I		4213	1.08
	Europe (non-Shengen)		5425	1.39
	Africa		4627	1.18
	Central/South America		12984	3.32
	Asia		1075	0.28
	Spain		362288	92.75
Sex	Male		216386	55.4
	Female		174226	44.6
Continuous variable	Min.–Max	Mean	Stand. dev. (overall)	N. of obs
Work experience	0–58	21.5	13.5785	389.019

## Results

**Fig. 1:**
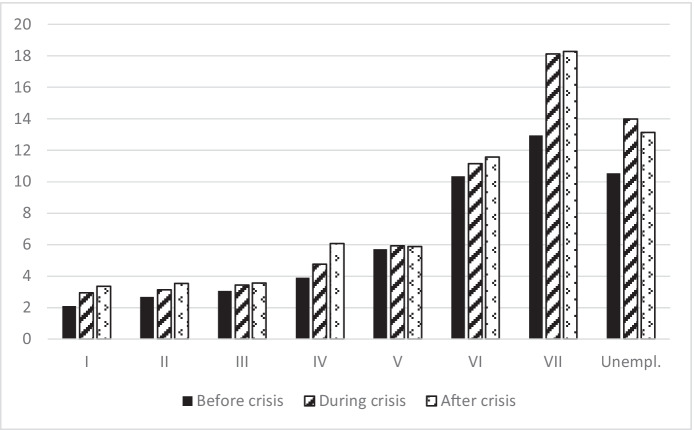
Percentage of migrants in each socio-occupational class before, during and after the Great Recession. Source: Own elaboration using Spanish Labour Force Survey, 2006–2016

Figure 1 presents the percentage of migrants in each socio-occupational class before, during and after the crisis. The figure clearly shows how the percentage of migrants is higher in the lowest socio-occupational categories. This ranges from 10.36% (before crisis) to 11.56% (after crisis) in the VI socio-occupational class, from 12.95% (before crisis) to 18.28% (after crisis) in the VII socio-occupational class and from 10.54% (before crisis) to 13.98% (during crisis) for unemployment. In contrast, the percentage of migrants in the three highest socio-occupational classes is marginal. This ranges from 3.05% (before crisis) to 3.58% (after crisis) in the III socio-occupational class, from 2.68% (before crisis) to 3.53% (after crisis) in the II socio-occupational class and from 2.1% (before crisis) to 3.35% (after crisis) in the I socio-occupational class. If we consider the entire time span observed, we can say that the distribution of migrants in the different socio-occupational classes during the observed periods appears to be quite stable over time, except for the last two socio-occupational classes, for which the percentage of migrant workers increased during and after the crisis. These results seem to confirm that migrants have a lower likelihood of belonging to higher socio-occupational classes than of belonging to the lowest socio-occupational classes or being unemployed (Tables 2, 3, 4, 5, 6, 7, 8, 9, and 10).

**Table 2:** Multinomial results predicting the relative risk of belonging to a socio-occupational class before, during and after crisis

Socio-occupational class *(Ref. III Intermediate professions)*		Before the crisis (2006–2008)			During the crisis (2010–2012)			After the crisis (2014–2016)		
I Higher grade university-level professionals and managers with 10 or more employees		RRR		SE	RRR		SE	RRR		SE
Origin *(ref. Spain)*	Europe I	1.22		0.17	1.16		0.13	1.37	**	0.15
	Europe (non-Shengen)	0.36	***	0.11	0.63	**	0.14	0.47	***	0.10
	Africa	0.55		0.25	0.74		0.25	0.85		0.31
	Central/South America	0.45	***	0.06	0.79	**	0.08	1.01		0.10
	Asia	0.55		0.26	0.50		0.23	0.52	+	0.20
Sex *(ref. Men)*	Women	0.55	***	0.01	0.70	***	0.02	0.78	***	0.02
Work experience		1.03	***	0.00	1.03	***	0.00	1.02	***	0.00
Educ. *(ref. Low)*	Middle	1.84	***	0.23	2.71	***	0.59	5.75	**	3.34
	Alto	75.87	***	9.54	171.51	***	37.20	603.80	***	349.66
_cons		0.02	***	0.00	0.01	***	0.00	0.00	***	0.00
II Lower grade university-level professionals and managers with less than 10 employees		RRR		SE	RRR		SE	RRR		SE
Origin *(ref. Spain)*	Europe I	1.65	***	0.19	1.40	**	0.15	1.43	***	0.15
	Europe (non-Shengen)	0.80		0.16	0.42	***	0.11	0.63	*	0.12
	Africa	1.46		0.35	1.01		0.26	0.68		0.23
	Central/South America	0.84	+	0.08	0.93		0.08	1.02		0.10
	Asia	1.45		0.39	1.35		0.30	0.89		0.21
*Sex (ref. Men)*	Women	0.35	***	0.01	0.39	***	0.01	0.50	***	0.01
Work experience		1.03	***	0.00	1.02	***	0.00	1.01	***	0.00
Educ. *(Ref. Low)*	Middle	0.86	***	0.04	0.70	***	0.03	0.86	+	0.07
	Alto	4.47	***	0.20	3.59	***	0.19	4.82	***	0.40
_cons		0.37	***	0.02	0.42	***	0.02	0.27	***	0.02
IV Small proprietors and self-employed workers		RRR		SE	RRR		SE	RRR		SE
Origin *(ref. Spain)*	Europe I	4.08	***	0.47	3.63	***	0.37	2.99	***	0.30
	Europe (non-Shengen)	1.93	**	0.41	2.33	***	0.45	1.50	*	0.26
	Africa	3.34	***	0.74	3.46	***	0.75	2.96	***	0.65
	Central/South America	1.75	***	0.19	1.94	***	0.18	2.33	***	0.20
	Asia	3.93	***	0.95	3.99	***	0.74	4.73	***	0.69
Sex *(ref. Men)*	Women	0.43	***	0.01	0.52	***	0.01	0.53	***	0.01
Work experience		1.04	***	0.00	1.04	***	0.00	1.03	***	0.00
Educ. *(ref. Low)*	Middle	0.33	***	0.01	0.29	***	0.01	0.26	***	0.01
	Alto	0.62	***	0.03	0.51	***	0.02	0.46	***	0.03
_cons		0.48	***	0.02	0.47	***	0.03	0.70	***	0.04
V Supervisors and workers in skilled technical occupations		RRR		SE	RRR		SE	RRR		SE
Origin *(ref. Spain)*	Europe I	1.15		0.15	1.43	**	0.17	1.23	+	0.15
	Europe (non-Shengen)	4.76	***	0.61	4.67	***	0.61	3.30	***	0.39
	Africa	2.49	***	0.45	2.55	***	0.47	3.13	***	0.58
	Central/South America	2.06	***	0.15	1.93	***	0.14	1.90	***	0.15
	Asia	0.44	**	0.14	0.14	***	0.06	0.23	***	0.06
Sex *(ref. Men)*	Women	0.12	***	0.00	0.12	***	0.00	0.13	***	0.00
Work experience		1.00	***	0.00	1.00		0.00	1.00		0.00
Educ. *(ref. Low)*	Middle	0.32	***	0.01	0.35	***	0.01	0.31	***	0.02
	Alto	0.06	***	0.00	0.06	***	0.00	0.04	***	0.00
_cons		4.69	***	0.19	3.16	***	0.15	3.79	***	0.21
VI Skilled and semi-skilled manual workers		RRR		SE	RRR		SE	RRR		SE
Origin *(ref. Spain)*	Europe I	2.03	***	0.23	1.86	***	0.18	1.61	***	0.16
	Europe (non-Shengen)	11.63	***	1.34	10.46	***	1.19	6.58	***	0.67
	Africa	6.12	***	1.01	5.61	***	0,93	4.90	***	0.84
	Centr/South America	4.51	***	0.28	4.42	***	0.25	4.42	***	0.28
	Asia	1.51	+	0.34	1.72	***	0.29	1.08		0.17
Sex *(ref. Men)*	Women	0.33	***	0.01	0.54	***	0.01	0.66	***	0.01
Work experience		1.01	***	0.00	1.01	***	0.00	1.00		0.00
Educ. *(ref. Low)*	Middle	0.27	***	0.01	0.26	***	0.01	0.24	***	0.01
	Alto	0.06	***	0.00	0.06	***	0.00	0.05	***	0.00
_cons		4.07	***	0.16	3,03	***	0.13	3.73	***	0.20
VII Unskilled manual workers		RRR		SE	RRR		SE	RRR		SE
Origin *(ref. Spain)*	Europe I	0.84		0.21	0.96		0,20	0,85		0,16
	Europe (non-Shengen)	9.00	***	1.31	10.54	***	1.45	9.58	***	1.11
	Africa	8.68	***	1.58	13.69	***	2.33	12.94	***	2.24
	Central/South America	3.48	***	0.32	4.56	***	0.35	4.29	***	0.35
	Asia	0.43	+	0.21	1.25		0.30	0.91		0.20
Sex *(ref. Men)*	Women	0.35	***	0.01	0.42	***	0.01	0.47	***	0.02
Work experience		0.98	***	0.00	0.98	***	0.00	1.00	***	0.00
Educ. *(ref. Low)*	Middle	0.13	***	0.01	0.12	***	0.01	0.10	***	0.01
	Alto	0,03	***	0.00	0.02	***	0.00	0.01	***	0.00
_cons		1.91	***	0.11	2.17	***	0.13	3.30	***	0.22
Unemployed		RRR		SE	RRR		SE	RRR		SE
Origin *(ref. Spain)*	Europe I	1.15		0.15	1.32	***	0.12	1.15		0.10
	Europe (non-Shengen)	5.05	***	0.61	6.62	***	0.72	4.08	***	0.39
	Africa	5.34	***	0.89	8.22	***	1.26	8.12	***	1.30
	Central/South America	2.24	***	0.15	2.75	***	0.15	2.91	***	0.17
	Asia	0.69		0.18	0.39	***	0.07	0.38	***	0.06
Sex *(ref. Men)*	Women	0.88	***	0.02	0.68	***	0.01	0.83	***	0.01
Work experience		0.97	***	0.00	0.97	***	0.00	0.97	***	0.00
Educ. *(ref. Low)*	Middle	0.14	***	0.00	0.13	***	0.00	0.11	***	0.00
	Alto	0.09	***	0.00	0.06	***	0.00	0.05	***	0.00
_cons		4.94	***	0.21	16.75	***	0.67	23.12	***	1.10
Pseudo *R*-squared		0.1384			0.1415			0.1425		
N. obs		123011			135630			130378		

+ *p* ≤ 0.1 * *p* ≤ 0.05, ** *p* ≤ 0.01, *** *p* ≤ 0.001

**Table 3:** Predictive margins for the first socio-occupational class: higher grade university-level professionals and managers with 10 or more employee

Before crisis	Margins (SE)	95% confidence interval		
Schengen EU + North America	0.068 (0.006)	0.05	-	0.07
Europe (Others)	0.020 (0.005)	0.01	-	0.03
Africa	0.027 (0.010)	0.01	-	0.48
Cent/South America	0.035 (0.004)	0.02	-	0.04
Asia	0.039 (0.015)	0.01	-	0.06
Spain	0.085 (0.000)	0.08	-	0.08
During crisis	Margins (SE)	95% confidence interval		
Schengen EU + North America	0.075 (0.005)	0.06	-	0.09
Europe (Others)	0.032 (0.006)	0.02	-	0.04
Africa	0.033 (0.009)	0.01	-	0.05
Cent/South America	0.056 (0.004)	0.05	-	0.06
Asia	0.043 (0.016)	0.01	-	0.07
Spain	0.091 (0.001)	0.08	-	0.09
After crisis	Margins (SE)	95% confidence interval		
Schengen EU + North America	0.091 (0.005)	0.08	-	0.1
Europe (Others)	0.032 (0.006)	0.02	-	0.04
Africa	0.038 (0.011)	0.02	-	0.06
Cent/South America	0.064 (0.004)	0.06	-	0.07
Asia	0.048 (0.014)	0.02	0	0.07
Spain	0.095 (0.001)	0.09	-	0.09

**Table 4:** Predictive margins for the second socio-occupational class: lower grade university-level professionals and managers with less than 10 employees

Before crisis	Margins (SE)	95% confidence interval		
Schengen EU + North America	0.146 (0.011)	0.13	-	0.17
Europe (Others)	0.051 (0.008)	0.04	-	0.07
Africa	0.094 (0.015)	0.07	-	0.12
Cent/South America	0.084 (0.006)	0.07	-	0.09
Asia	0.166 (0.028)	0.11	-	0.22
Spain	0.141 (0.001)	0.14	-	0.14
During crisis	Margins (SE)	95% confidence interval		
Schengen EU + North America	0.106 (0.007)	0.09	-	0.12
Europe (Others)	0.019 (0.004)	0.01	-	0.03
Africa	0.042 (0.008)	0.03	-	0.06
Cent/South America	0.064 (0.004)	0.06	-	0.07
Asia	0.138 (0.021)	0.09	-	0.18
Spain	0.112 (0.001)	0.11	-	0.11
After crisis	Margins (SE)	95% confidence interval		
Schengen EU + North America	0.094 (0.007)	0.08	-	0.12
Europe (Others)	0.032 (0.005)	0.02	-	0.04
Africa	0.024 (0.007)	0.01	-	0.04
Cent/South America	0.053 (0.004)	0.04	-	0.06
Asia	0.079 (0.091)	0.05	-	0.11
Spain	0.091 (0.001)	0.09	-	0.09

**Table 5:** Predictive margins for the third socio-occupational class: intermediate professions—administrative employees and administrative management and other service support professionals

Before crisis	Margins (SE)	95% confidence interval		
Schengen EU + North America	0.149 (0.011)	0.13	-	0.17
Europe (Others)	0.074 (0.007)	0.06	-	0.09
Africa	0.085 (0.012)	0.06	-	0.11
Cent/South America	0.130 (0.006)	0.12	-	0.14
Asia	0.189 (0.027)	0.14	-	0.24
Spain	0.226 (0.001)	0.22	-	0.23
During crisis	Margins (SE)	95% confidence interval		
Schengen EU + North America	0.142 (0.009)	0.13	-	0.16
Europe (Others)	0.061 (0.006)	0.04	-	0.07
Africa	0.060 (0.008)	0.04	-	0.07
Cent/South America	0.106 (0.005)	0.09	-	0.12
Asia	0.194 (0.022)	0.15	-	0.24
Spain	0.209 (0.001)	0.21	-	0.21
After crisis	Margins (SE)	95% confidence interval		
Schengen EU + North America	0.149 (0.009)	0.13	-	0.16
Europe (Others)	0.082 (0.007)	0.07	-	0.09
Africa	0.058 (0.009)	0.04	-	0.08
Cent/South America	0.097 (0.004)	0.09	-	0.1
Asia	0.205 (0.019)	0.17	-	0.24
Spain	0.203 (0.001)	0.2	-	0.21

**Table 6:** Predictive margins for the fourth socio-occupational class: small proprietors and self-employed workers

Before crisis	Margins (SE)	95% confidence interval		
Schengen EU + North America	0.211 (0.014)	0.18	-	0.24
Europe (Others)	0.051 (0.009)	0.03	-	0.07
Africa	0,102 (0.015)	0.07	-	0.13
Cent/South America	0.082 (0.007)	0.07	-	0.09
Asia	0.260 (0.034)	0.19	-	0.33
Spain	0.086 (0.001)	0.08	-	0.09
During crisis	Margins (SE)	95% confidence interval		
Schengen EU + North America	0.18 (0.011)	0.16	-	0.2
Europe (Others)	0.052 (0.008)	0.04	-	0.07
Africa	0.078 (0.011)	0.06	-	0.09
Cent/South America	0.075 (0.005)	0.06	-	0.09
Asia	0.264 (0.027)	0.21	-	0.32
Spain	0.078 (0.001)	0.07	-	0.08
After crisis	Margins (SE)	95% confidence interval		
Schengen EU + North America	0.170 (0.010)	0.15	-	0.19
Europe (Others)	0.050 (0.007)	0.04	-	0.06
Africa	0.070 (0.010)	0.05	-	0.09
Cent/South America	0.089 (0.001)	0.08	-	0.09
Asia	0.358 (0.024)	0.31	-	0.4
Spain	0.081 (0.001)	0.08	-	0.08

**Table 7:** Predictive margins for the fifth socio-occupational class: supervisors and workers in skilled technical occupations

Before crisis	Margins (SE)	95% confidence interval		
Schengen EU + North America	0.102 (0.009)	0.08	-	0.12
Europe (Others)	0.142 (0.009)	0.13	-	0.16
Africa	0.104 (0.009)	0.09	-	0.12
Cent/South America	0.130 (0.006)	0.12	-	0.14
Asia	0.052 (0.013)	0.03	-	0.08
Spain	0.143 (0.001)	0.14	-	0.14
During crisis	Margins (SE)	95% confidence interval		
Schengen EU + North America	0.095 (0.008)	0.8	-	0.11
Europe (Others)	0.099 (0.007)	0.9	-	0.11
Africa	0.060 (0.006)	0.05	-	0.07
Cent/South America	0.084 (0.004)	0.08	-	0.09
Asia	0.013 (0.005)	0.01	-	0.02
Spain	0.103 (0.001)	0.1	-	0,1
After crisis	Margins (SE)	95% confidence interval		
Schengen EU + North America	0.091 (0.008)	0.08	-	0.1
Europe (Others)	0.098 (0.007)	0.08	-	0.11
Africa	0.071 (0.006)	0.06	-	0.08
Cent/South America	0.078 (0.004)	0.07	-	0.09
Asia	0.024 (0.006)	0.01	-	0.04
Spain	0.101 (0.001)	0.09	-	0.1

**Table 8:** Predictive margins for the sixth socio-occupational class: skilled and semi-skilled manual workers

Before crisis	Margins (SE)	95% confidence interval		
Schengen EU + North America	0.223 (0.014)	0.19	-	0.25
Europe (Others)	0.453 (0.013)	0.43	-	0.48
Africa	0.322 (0.015)	0.29	-	0.35
Cent/South America	0.364 (0.009)	0.35	-	0.38
Asia	0.217 (0.026)	0.17		0.27
Spain	0.178 (0.001)	0.17	-	0.18
During crisis	Margins (SE)	95% confidence interval		
Schengen EU + North America	0.170 (0.010)	0.15	-	0.19
Europe (Others)	0.316 (0.011)	0.29	-	0.34
Africa	0.183 (0.011)	0.16	-	0.21
Cent/South America	0.269 (0.007)	0.26	-	0.28
Asia	0.220 (0.020)	0.18	-	0.26
Spain	0.141 (0.001)	0.14	-	0.14
After crisis	Margins (SE)	95% confidence interval		
Schengen EU + North America	0.154 (0.009)	0.13	-	0.17
Europe (Others)	0.261 (0.009)	0.24	-	0.28
Africa	0.144 (0.009)	0.13	-	0.16
Cent/South America	0.235 (0.006)	0.22	-	0.25
Asia	0.143 (0.014)	0.12	-	0.17
Spain	0.129 (0.001)	0.13	-	0.13

**Table 9:** Predictive margins for the seventh socio-occupational class: unskilled manual workers

Before crisis	Margins (SE)	95% confidence interval		
Schengen EU + North America	0.167 (0.004)	0.01	-	0.02
Europe (Others)	0.060 (0.006)	0.05	-	0.07
Africa	0.078 (0.007)	0.06	-	0.09
Cent/South America	0.049 (0.003)	0.04	-	0.05
Asia	0.011 (0.005)	0.01	-	0.02
Spain	0.032 (0.001)	0.03	-	0.03
During crisis	Margins (SE)	95% confidence interval		
Schengen EU + North America	0.184 (0.003)	0.12	-	0.25
Europe (Others)	0.062 (0.005)	0.05	-	0.07
Africa	0.086 (0.006)	0.07	-	0.09
Cent/South America	0.055 (0.003)	0.04	-	0.06
Asia	0.035 (0.007)	0.02	-	0.05
Spain	0.029 (0.001)	0.03	-	0.03
After crisis	Margins (SE)	95% confidence interval		
Schengen EU + North America	0.021 (0.003)	0.14	-	0.28
Europe (Others)	0.092 (0.006)	0.08	-	0.1
Africa	0.092 (0.006)	0.08	-	0.1
Cent/South America	0.057 (0.003)	0.05	-	0.06
Asia	0.032 (0.006)	0.02	-	0.04
Spain	0.337 (0.001)	0.032	-	0.034

**Table 10:** Predictive margins for the unemployment

Before crisis	Margin(SE)	95% Confidence Interval		
Schengen EU + North America	0.083 (0.007)	0,07	-	0,09
Europe (Others)	0.147 (0.008)	0,13	-	0,16
Africa	0.188 (0.012)	0,17	-	0,21
Cent/South America	0.125 (0.005)	0,12	-	0,13
Asia	0.065 (0.012)	0,04	-	0,09
Spain	0.109 (0.001)	0,11	-	0,11
During crisis	Margin(SE)	95% Confidence Interval		
Schengen EU + North America	0.211 (0.001)	0.19	-	0.23
Europe (Others)	0.365 (0.010)	0.34	-	0.38
Africa	0.456 (0.137)	0.43	-	0.48
Cent/South America	0.290 (0.006)	0.28	-	0.3
Asia	0.092 (0.011)	0.07	-	0.11
Spain	0.237 (0.001)	0.24	-	0.24
After crisis	Margin(SE)	95% Confidence Interval		
Schengen EU + North America	0.229 (0.009)	0.21	-	0.25
Europe (Others)	0.353 (0.010)	0.33	-	0.37
Africa	0.501 (0.014)	0.47	-	0.53
Cent/South America	0.326 (0.007)	0.31	-	0.33
Asia	0.111 (0.011)	0.09	-	0.13
Spain	0.267 (0.001)	0.26	-	0.27

**Fig. 2:**
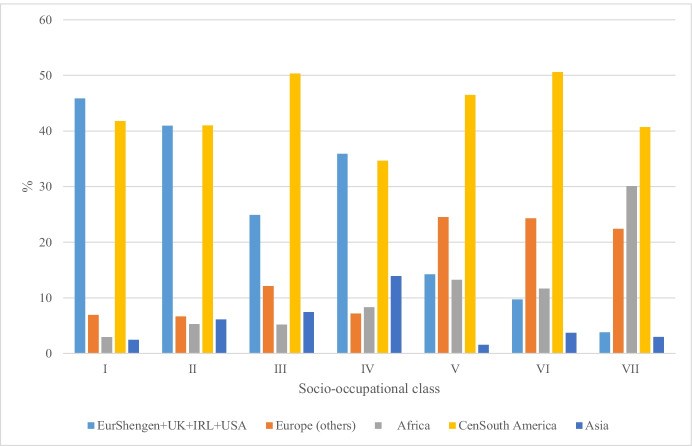
Segmentation of the Spanish labour market for the period 2006–2016 according to nationality. Source: Own elaboration using Spanish Labour Force Survey, 2006–2016

The results by migrants’ origin are displayed in Fig. 2. The figure shows how immigrants from Africa and Eastern Europe are almost non-present in the highest socio-occupational classes. On the other hand, Europeans from the Schengen area, Canadians and North Americans are located mainly in the higher socio-occupational classes compared with migrants from other origins. People from Latin American countries are represented in all socio-occupational classes and seem to be the collective with the greatest socio-occupational integration. Finally, the vast majority of workers of Asian origin work on their own.

As highlighted by the literature review, other variables, such as the amount of time spent in the Spanish labour market or the level of education, could be a factor and could determine a worse occupational outcome for migrants and, among them, certain categories of migrants. Therefore, it is important to further explore these results with a multivariate analysis that allows these variables to be taken into account. To test our hypotheses and to predict migrants’ probabilities of belonging to a certain socio-occupational class before, during and after the Great Recession by place of origin, we performed three multinomial logit regressions for each of the three periods under analysis. Firstly, for each migrant group (according to the variable “origin”), we calculated the relative risk ratios (RRR) of belonging to each socio-occupational category (over the reference category: the third socio-occupational class), compared to the natives. Secondly, to facilitate the interpretation of the results concerning the probabilities of belonging to each socio-occupational class by area of origin in each period under analysis, we calculated predictive margins. To do so, we varied the characteristic of interest (*origin*) across the whole dataset, while maintaining other characteristics constant, and we averaged the predictions for each outcome. The differences in the sets of calculated probabilities can be interpreted as the difference due to the variable of interest (*origin*), as all other characteristics are maintained constant.

For the sake of clarity, we chose to display the results of the predictive margins graphically. Tabular presentations of the results displayed in the figures, as well as the tabular presentation of the full regressions showing the RRR for all the variables, can be found in the Annexes. In the following eight figures, we plot the predicted margins’ results for each socio-occupational class for the three periods together. The dots indicate the point estimates, whereas the lines show 95% confidence intervals.

**Fig. 3:**
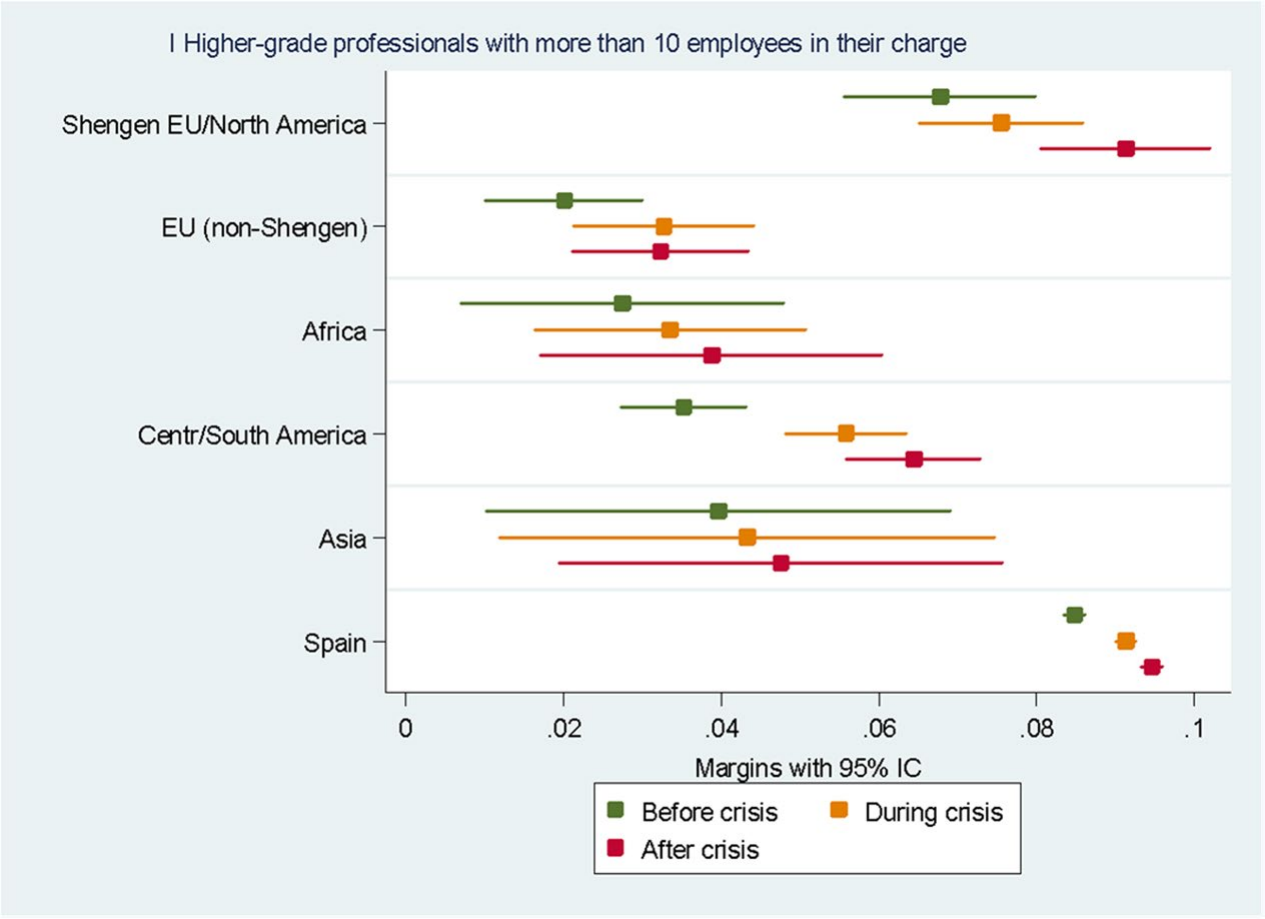
Predictive margins of being in the first socio-occupational class. Source: Own elaboration using Spanish Labour Force Survey, 2006–2016. For the tabular presentation of results, see Table 3

Figure 3 displays the results for the I socio-occupational class “higher grade university-level professionals and managers with 10 or more employees”. We can see how, before the crisis, natives had a higher probability (average probability: 8.5%) of belonging to this class compared to migrants. Among migrants, Europeans from the Schengen areas, British and North Americans were the best positioned in the socio-occupational structure. On average, their probability of belonging to this socio-occupational class was 6.8%. The migrants’ groups with the lowest probability of belonging to this socio-occupational class were Europeans from outside the Schengen Area, with an average probability of 2%, and Africans, with an average probability of 2.7%. Interestingly, if we look at differences across the three periods, the likelihood of belonging to this class increased during and after the crisis for natives, for Europeans from the Schengen areas and North Americans, and for people from Central and South America. In the case of Europeans from the Schengen areas, British and North Americans, their likelihood of belonging to the first socio-occupational class (average probability: 9.1%) after the crisis seems to no longer be significantly different from that of natives (average probability: 9.4%). Even though people from Central and South America still have lower probabilities of belonging to this class than natives, they are the migrant group for whom the increase in the likelihood of belonging to this class increased the most after the crisis: from an average probability of 3.5% before the crisis to 6.4% in the post-crisis period. No significant difference can be appreciated for the rest of the groups.

**Fig. 4:**
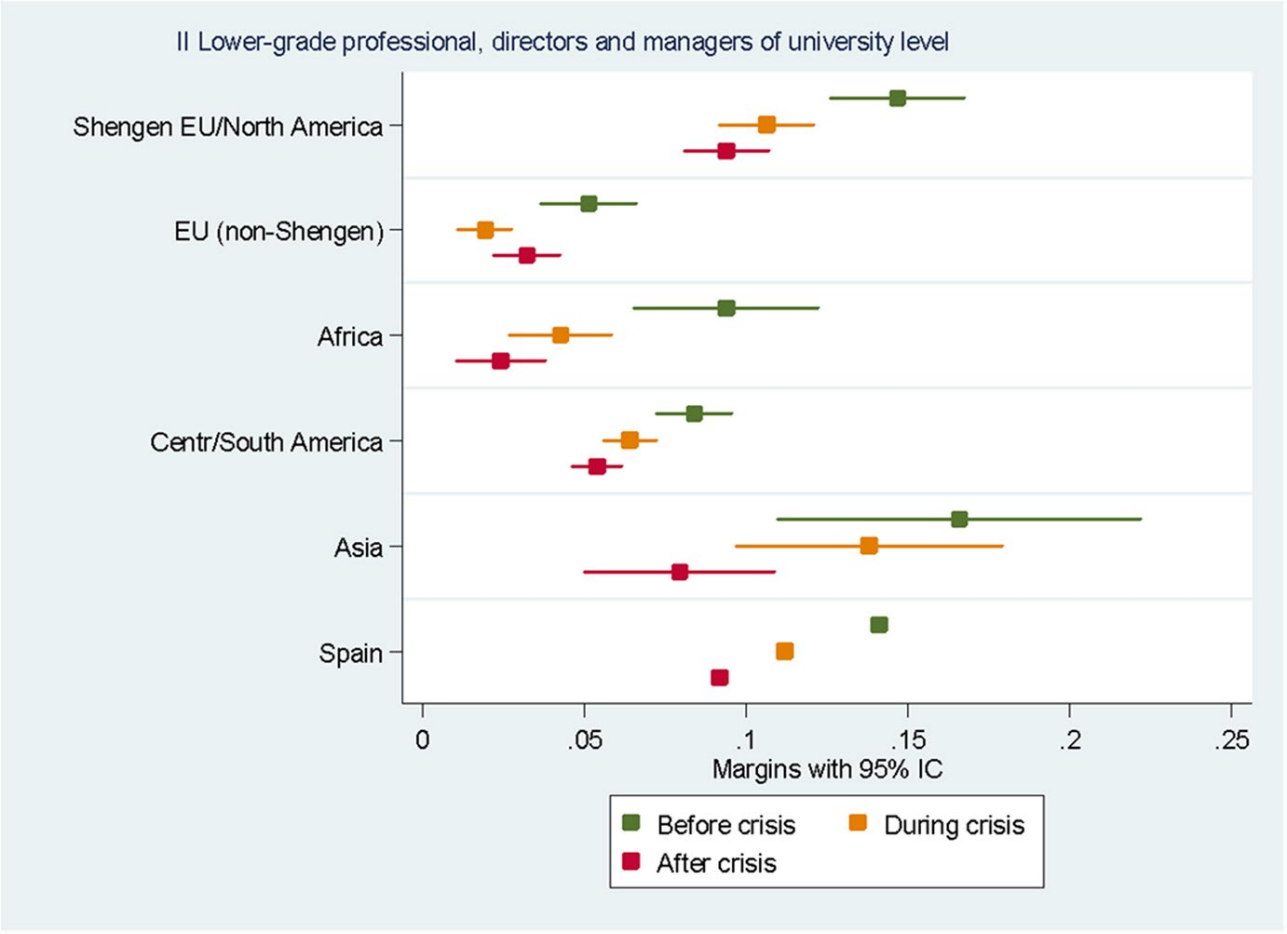
Predictive margins of being in the second socio-occupational class. Source: Own elaboration using Spanish Labour Force Survey, 2006–2016. For the tabular presentation of results, see Table 4

The results for the II socio-occupational class “lower grade university-level professionals and managers with less than 10 employees”, displayed in Fig. 4, show how no difference between natives and migrants from the Schengen area, Britain and North America and Asia can be found. The probability of a native belonging to this socio-occupational class before the crisis was on average 14.1%. For migrants from the Schengen area, Britain and North America, it was on average 14.7%, whereas for Asians it was 16.6%. Similarly to the first socio-occupational class, Europeans from outside the Schengen Area, British and North Americans were the migrants’ group with the lowest probability of belonging to this class, with an average probability of 5.1%. Contrary to what happens with the first socio-occupational class, the probabilities of belonging to the second social class decreased for all groups during the crisis and even more after it.

**Fig. 5:**
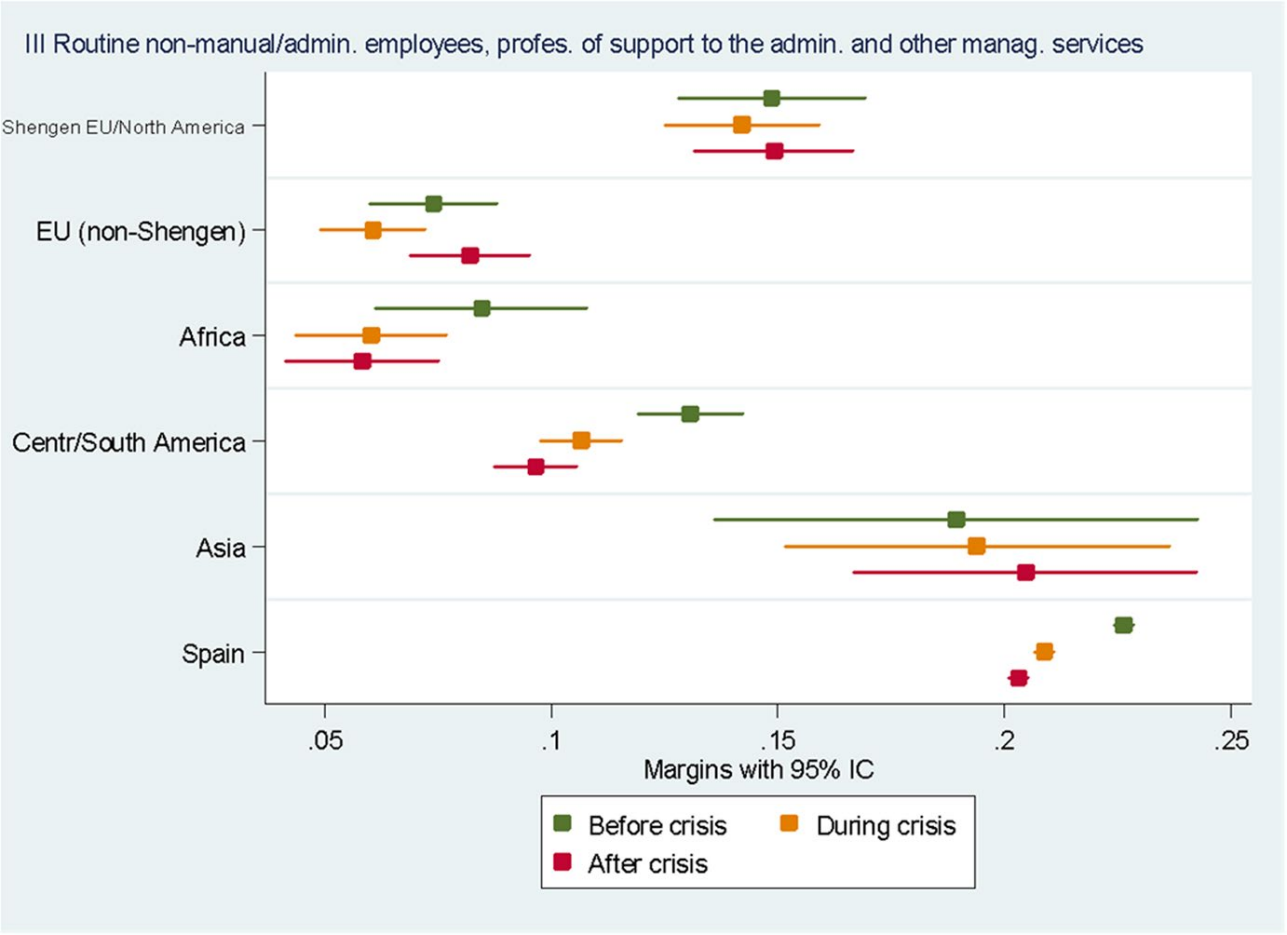
Predictive margins of being in the third socio-occupational class. Source: Own elaboration using Spanish Labour Force Survey, 2006–2016. For the tabular presentation of results, see Table 5

Figure 5 shows the results obtained for the III socio-occupational class “Intermediate professions: administrative employees and administrative management and other service support professionals”. Here again, the differences between natives and migrants are clear. Whereas, before the crisis, the likelihood of belonging to this socio-occupational class was on average 22.6% for natives, it was only 14.9% for Europeans from the Schengen area, British and North Americans, which constituted the migrant group with the highest probability of belonging to this socio-occupational class, followed by people from Central and South America. The results obtained for Asians are difficult to interpret since they seem to concern a very small and heterogeneous group, as indicated by the very long confidence interval lines. The difference between natives and migrants in this class may be due to the fact that this class includes a number of professions in the public sector. Looking at changes over time, we can see that the likelihood of belonging to this class decreased during the crisis for Spaniards and people from Central and South America and remained stable after the crisis. For all the other groups no significant differences in the three periods can be appreciated.

**Fig. 6:**
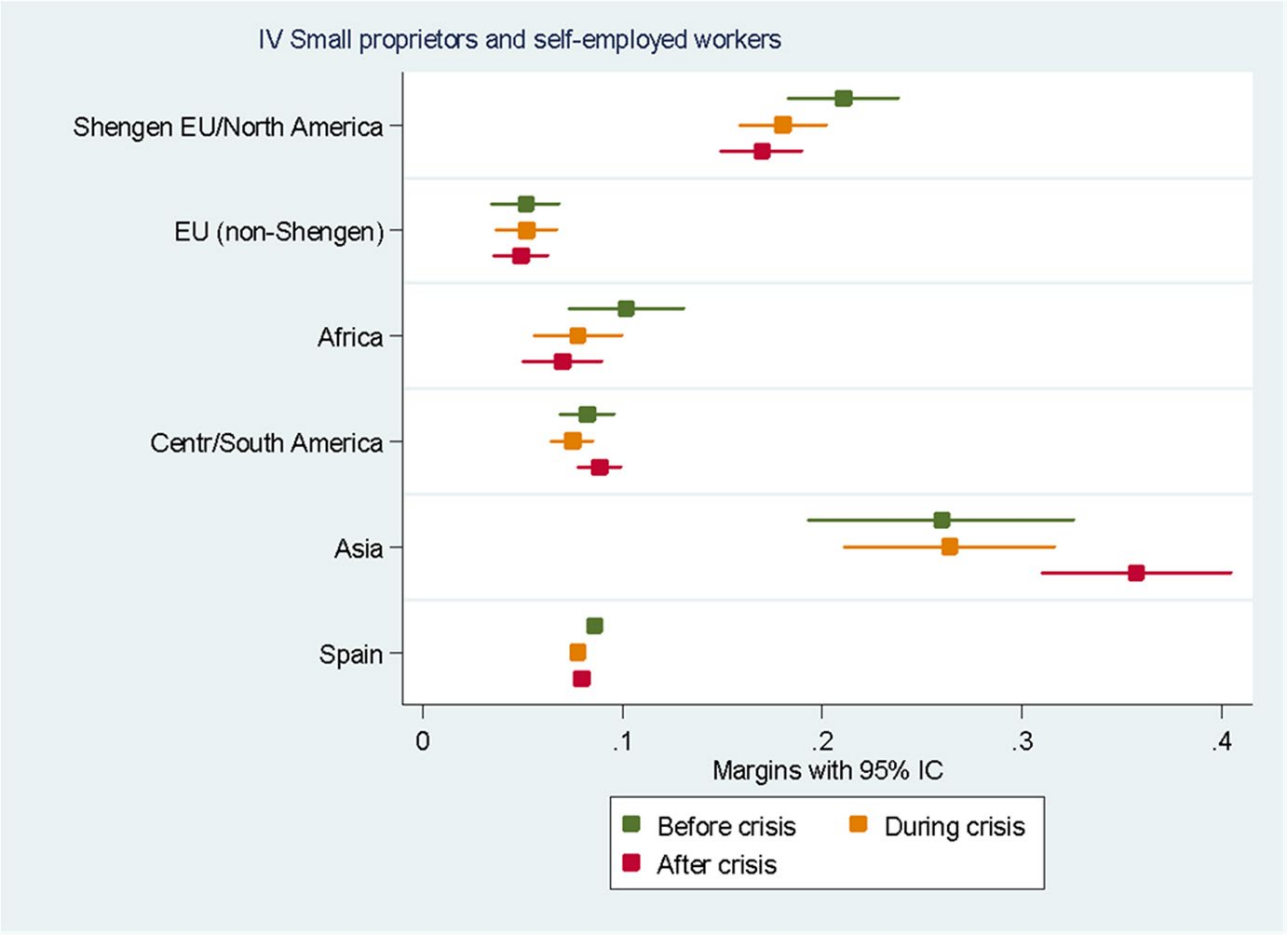
Predictive margins of being in the fourth socio-occupational class. Source: Own elaboration using Spanish Labour Force Survey, 2006–2016. For the tabular presentation of results, see Table 6

The results concerning the IV socio-occupational class “Small proprietors and self-employed workers”, displayed in Fig. 6, show how in the whole period under analysis Asians and Europeans from the Schengen Area, British and North Americans had higher probabilities of being small proprietors and self-employed than natives, with an average probability of 25.9% and 21%, respectively. The average probability for Spaniards was only 8.6%. For Asians, the likelihood of belonging to this class remained stable during the crisis and significantly increased after it. For all the other groups, including natives, the likelihood of belonging to this class slightly decreased during the crisis and remained almost stable after that.

**Fig. 7:**
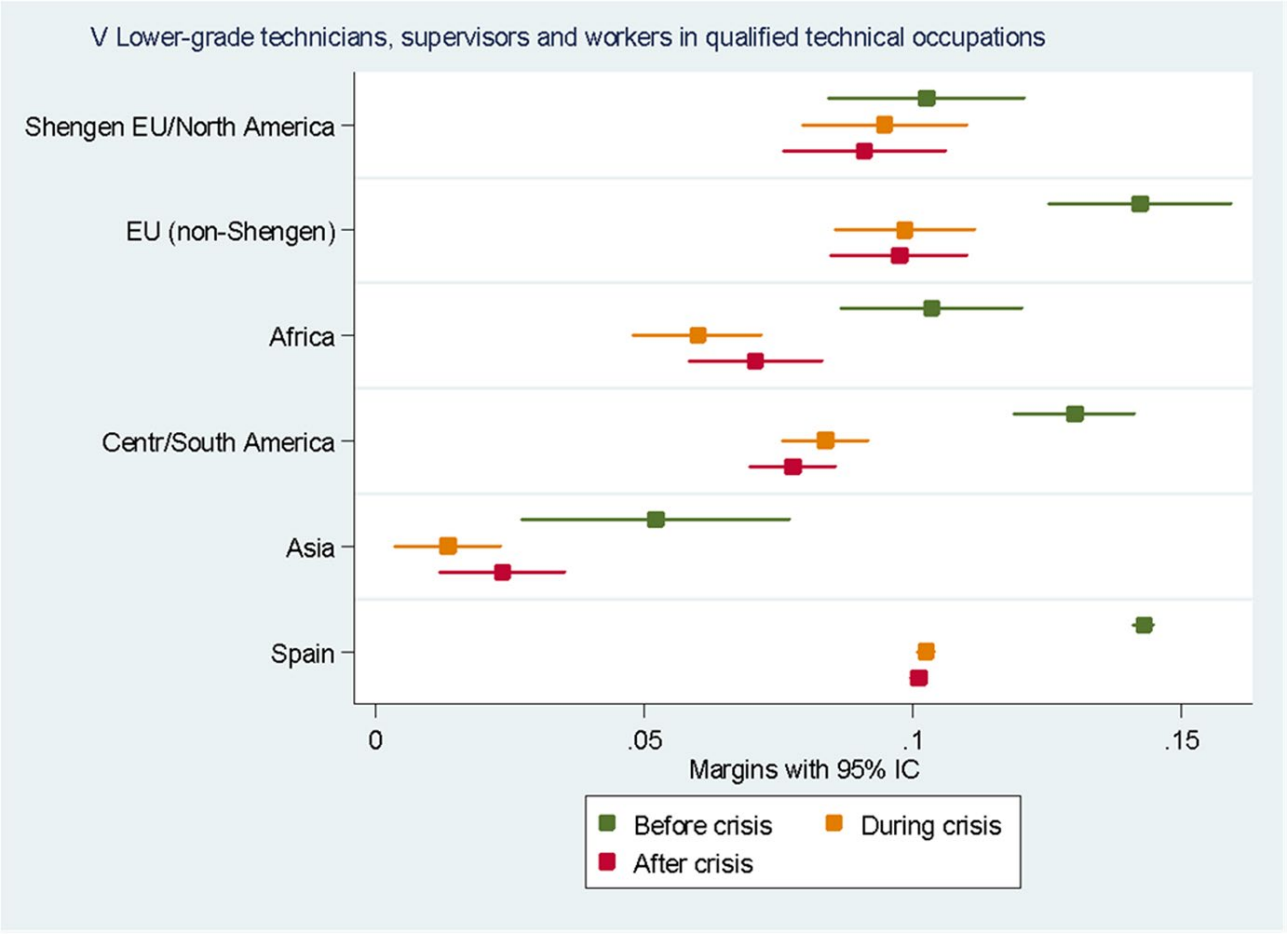
Predictive margins of being in the fifth socio-occupational class. Source: Own elaboration using Spanish Labour Force Survey, 2006–2016. For the tabular presentation of results, see Table 7

Looking at the V class “Supervisors and workers in skilled technical occupations”, in Fig. 7, we can see that the two groups of migrants most likely to belong to this class, with a likelihood that appears equal to that of natives, are Europeans from outside the Schengen Area and people from Central and South America. The likelihood of belonging to this class decreased for all the groups during the crisis and remained stable after that. The decrease was particularly pronounced for people from Central and South America: their likelihood of belonging to this class decreased from an average probability of 13% before the crisis to 7.8% after it.

**Fig. 8:**
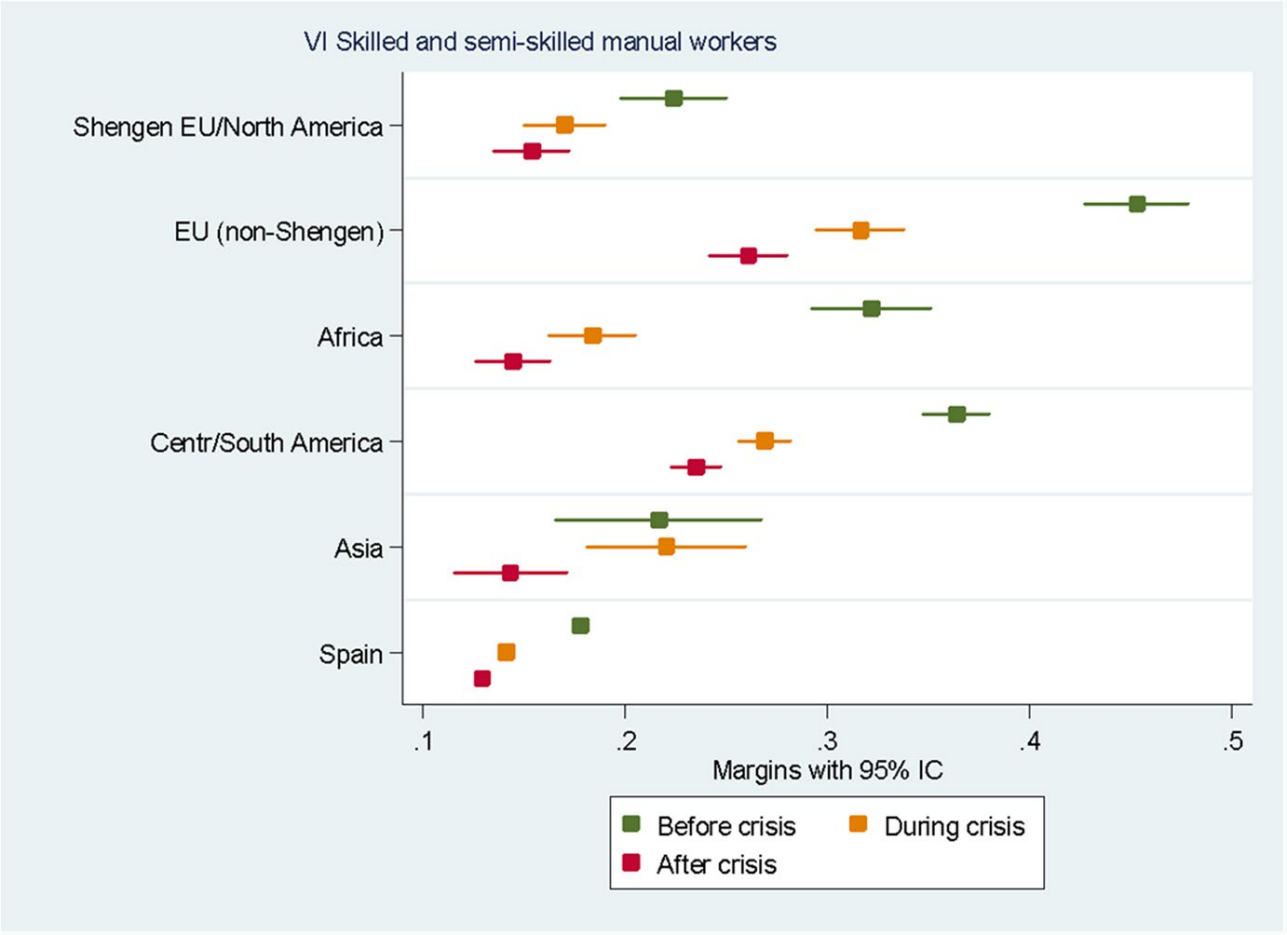
Predictive margins of being in the sixth socio-occupational class. Source: Own elaboration using Spanish Labour Force Survey, 2006–2016. For the tabular presentation of results, see Table 8

Figure 8 concerns the VI class “Skilled and semi-skilled manual workers”. Before the crisis, we can see how Europeans from non-Schengen countries, individuals from Central and South America, and Africans had the highest probability of belonging to this class. During the crisis, and even more after it, the likelihood of belonging to this socio-occupational class decreased for all groups, even though individuals from non-Schengen countries and from Central and South America continued to be the two groups with the highest probability of belonging to this class even after the crisis. The probability of a native belonging to this class was very low before the crisis (average probability: 1.8%) and became even lower during the crisis (average probability: 1.4%) and after it (average probability: 1.3%).

**Fig. 9:**
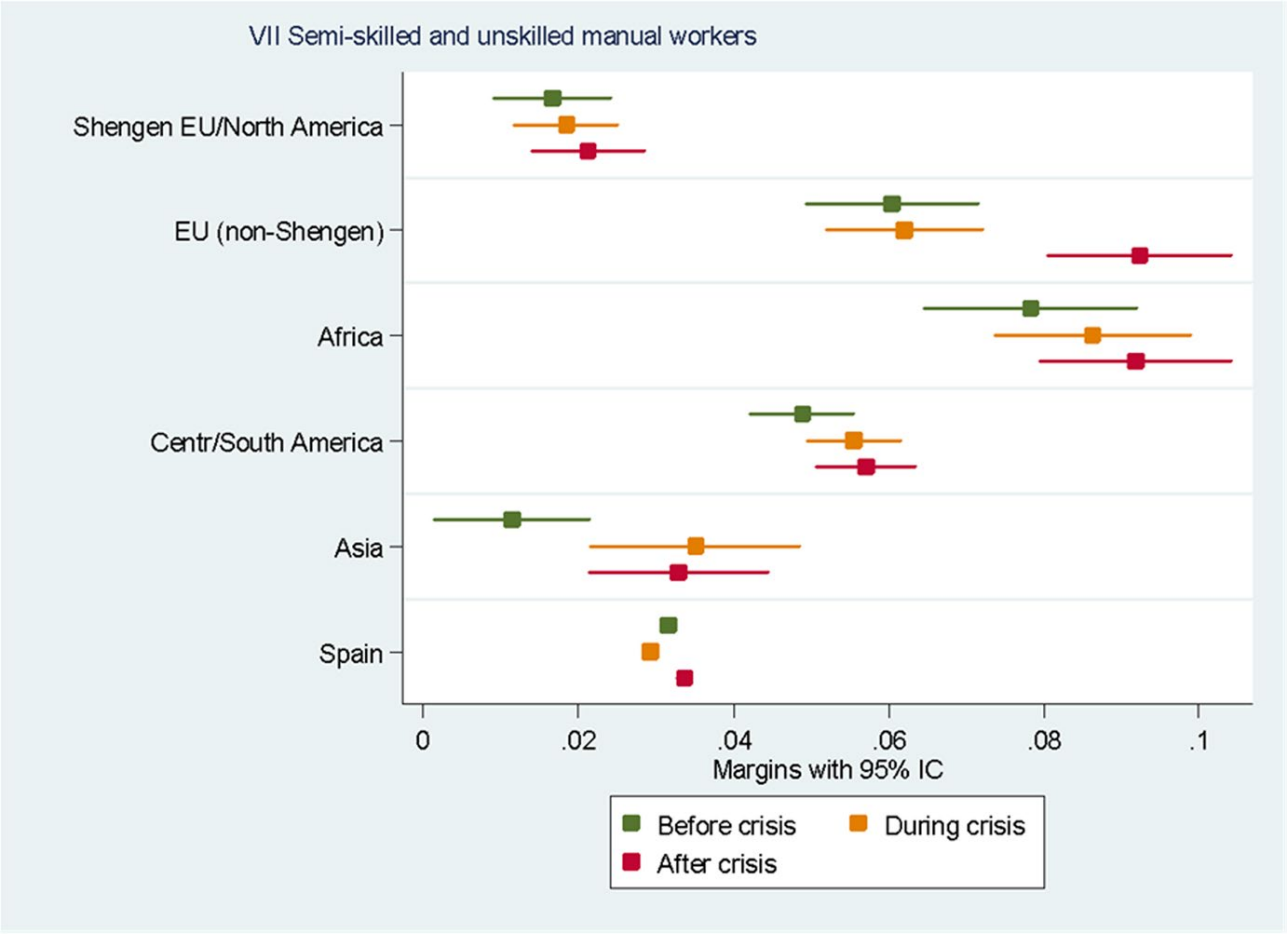
Predictive margins of being in the seventh socio-occupational class. Source: Own elaboration using Spanish Labour Force Survey, 2006–2016. For the tabular presentation of results, see Table 9

Figure 9 shows that Africans are the group with the highest likelihood of belonging to class VII “Unskilled manual workers” in all the three periods observed, followed by Europeans from non-Schengen countries and individuals from Central and South America. If we look at the results for the crisis period, we can observe a slight increase in the likelihood of belonging to this class for all the groups, but this increase does not seem to be significant, with the exception of Asians, whose likelihood of belonging to this class increased significantly during the crisis and remained stable after that. For non-Schengen Europeans, the probability of belonging to this class increased significantly after the crisis and became more similar to that of Africans.

**Fig. 10:**
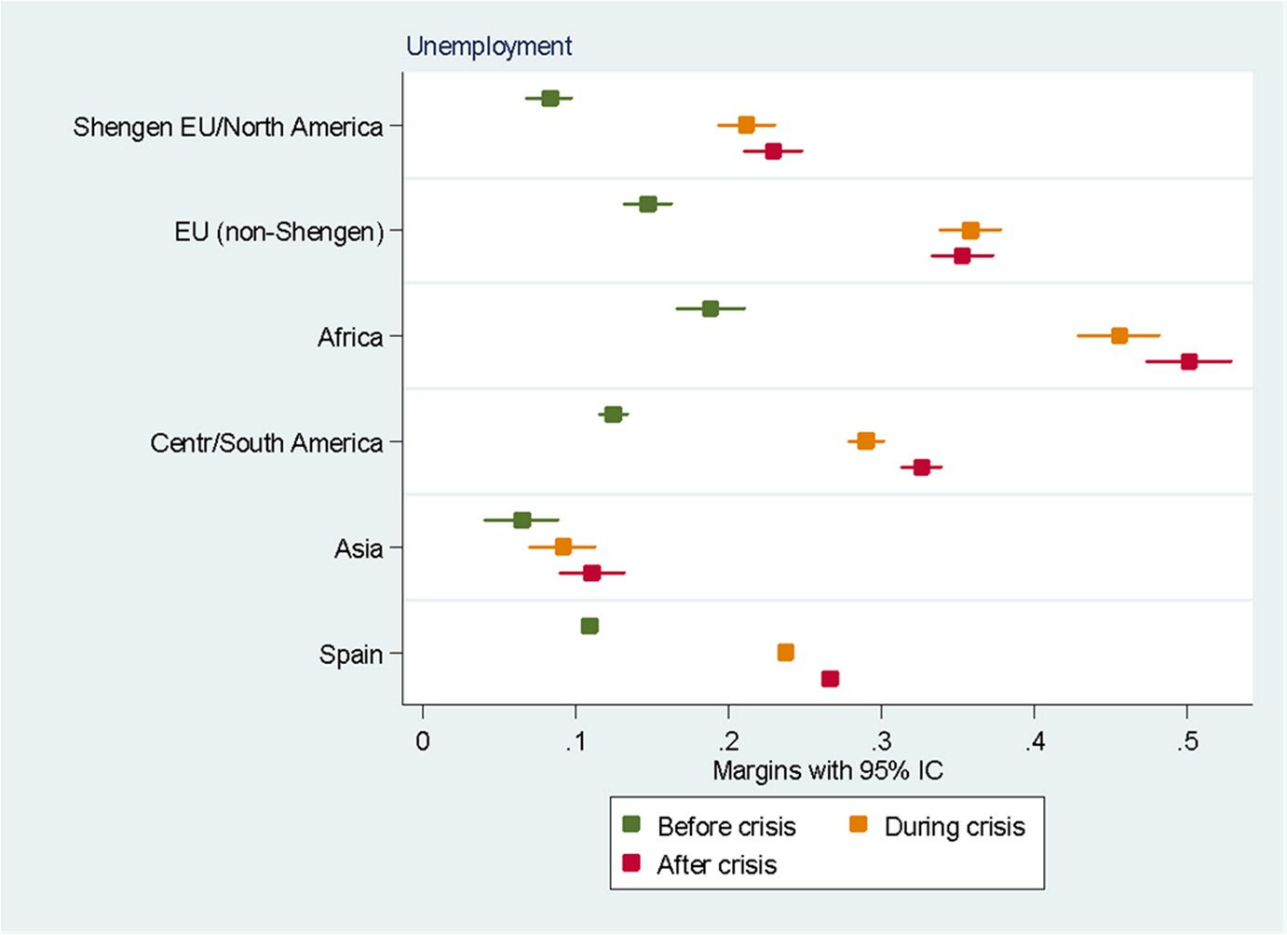
Predictive margins of being unemployed. Source: Own elaboration using Spanish Labour Force Survey, 2006–2016. For the tabular presentation of results, see Table 10

As can be noted in Fig. 10, Africans are the most likely to be unemployed compared to other groups in the three periods observed, followed by Non-Schengen Europeans and Central and South Americans. Interestingly, before the crisis, the likelihood of being unemployed for Asians and Europeans from the Schengen area and North Americans was lower than that of natives. During the crisis, the probabilities of being unemployed dramatically increased for all the groups, with the exception of Asians. Finally, the results after the crisis show a worrying trend for almost all groups. Despite the economic recovery in Spain after the crisis, the chances of being unemployed remained stable at the levels they had shown during the crisis for Asians and Europeans from the Schengen area and North Americans, and even increased for the rest of the groups.

## Discussion

### Natives and Migrants in the Spanish Socio-occupational Structure

The results confirm an intense segmentation of the Spanish labour market, with natives generally having higher likelihoods of belonging to the first three socio-occupational classes than migrants. However, we can only partially confirm our first hypothesis (H1), according to which we expected that, even after controlling for educational level and work experience, compared to natives, migrants would have a lower likelihood of belonging to higher socio-occupational classes and a higher likelihood of belonging to the lowest socio-occupational classes or being unemployed (“ethnic penalty”). Indeed, this is true only for some groups of migrants. The results show, for example, that, in all three periods, the probability of natives belonging to the first two socio-occupational classes is very similar to that of Europeans from the Schengen Area, British and North Americans.

### Distribution of Migrants in the Socio-occupational Structure According to the Place of Origin

The results show a very unequal distribution of immigrants in the socio-occupational structure, varying according to their origin, even when maintaining crucial variables such as educational level and years of experience constant. This confirms our second hypothesis (H2) that a migrant’s likelihood of belonging to a certain socio-occupational class will differ according to the migrant’s origin with some groups displaying a larger “ethnic penalty” than others (“ethnic-stratified labour market”). The origin of migrants is thus crucial, even after controlling for years of experience in the Spanish labour market and educational level. While migrants from impoverished countries are over-represented in the lower socio-occupational classes, migrants from enriched countries (Europeans from Schengen countries, British and North Americans) have similar probabilities as natives of accessing the best occupations in the three periods. This beneficial situation for this group of migrants could be related to the policies of degree recognition existing at the European level and with some countries, such as the USA or Canada. Nevertheless, it could also be due to the free movement space and work visa facilitations they enjoy in comparison with other migrants. Finally, it could be that, coming from enriched countries, they are less vulnerable in the labour market. Concerning Latin American migrants, even though their likelihood of belonging to the first socio-occupational categories is lower than that of natives, they seem to be “integrated” in all the socio-occupational classes, probably because of their language knowledge, confirming the results of Cebolla-Boado et al. (2015). According to Solé and Parella (2003), Latin-Americans enjoy better social acceptance in Spain because of their cultural proximity with natives, which makes their labour participation easier. The “ethnic penalty” in Spain seems to particularly concern Africans and Europeans from non-Schengen countries, which are the two worst-situated groups in the Spanish labour market and also the ones most likely to be unemployed before the crisis. Various mechanisms could explain this result. Firstly, these inequalities may be the result of employer discrimination and prejudice against some particular groups of migrants (see, e.g., Pager & Quilian, 2005). As access to jobs is often mediated by social networks, this can lead to the creation of ethnic niches. Secondly, they can also relate to the particularly vulnerable situation experienced by some migrants. Whereas Europeans from the Schengen area do not need to work to be allowed to stay in Spain and have no difficulties in maintaining contact with families and friends in their countries of origin, and whereas migrants from Central and Southern America and Asians benefit from larger networks of compatriots in Spain, Africans and Eastern Europeans are newcomers in Spain and often lack these kinds of support. They therefore may have a more urgent need to work, which causes them to accept jobs for which they may be overqualified. For their parts, Asians constitute a singular case: they are the group that is least likely to be unemployed and the most likely to be self-employed. This tendency of Asians to be dedicated to commercial and entrepreneurial activities has already been highlighted by previous research (e.g., Arjona & Checa-Olmos, 2006).

### Evolution over Time: Migrants in the Spanish Labour Market Before, During and After the Great Recession

If we look at the results concerning the crisis and the post-crisis periods, we observe that, compared to the before-crisis period, the likelihood of all groups of belonging to the IV socio-occupational class “Small proprietors and self-employed workers” remained stable during the crisis. In the post-crisis period, Asians were even more likely to be represented in this category. Instead, the likelihood of belonging to all intermediate socio-occupational classes, from II “Lower grade university-level professionals and managers with less than 10 employees” to VI “Skilled and semi-skilled manual workers”, decreased for all groups during the crisis. The probability of being employed in these professions was generally even lower after the crisis. This tendency seems to have not affected the VII socio-occupational class (“Unskilled manual workers”). Indeed, the probability of belonging to this socio-occupational class increased slightly during and after the crisis for all the groups that already had comparative higher probabilities of belonging to this socio-occupational class. This is perhaps due to the increased vulnerability that some groups have experienced due to the implementation of austerity policies and the cuts in social rights. In other words, as they are more vulnerable, they are more dependent on income derived from work and therefore prefer to work even if it is in the last social class rather than being unemployed (Bruquetas-Callejo & Moreno, 2015; Pavolini et al., 2015). The results concerning unemployment show how the probability of being unemployed was higher during the crisis than before it and that, after the crisis, it was even higher than during the crisis for almost all groups. These results confirm our hypothesis (H3) regarding an increased likelihood of unemployment for both natives and migrants during and after the crisis compared to the before-crisis period and a decreased likelihood for both natives and migrants of belonging to a middle socio-occupational class (“job polarisation”), confirming the results of previous research (Bisello et al., 2020). The destruction of employment in the middle socio-occupational classes seems to have corresponded to a downgrade of the working conditions of many workers. It seems that this destruction of jobs in the middle socio-occupational classes may have gone to fuel long-term unemployment (Bisello et al., 2020). Interestingly, in contrast, the likelihood of belonging to the I socio-occupational class “Higher grade university-level professionals and managers with 10 or more employees” was higher during the crisis, and even higher after it. This increase concerned the groups that already had comparatively higher probabilities of belonging to this socio-occupational class before the crisis (natives, migrants from Central and South America, Schengen countries, the UK and North America). All these results confirm a tendency towards an increased job polarisation due to the Great Recession, i.e. the decline of mid-paid jobs with respect to jobs at the top and bottom of the labour market structure (Fernández-Macías & Hurley, 2017), which was already found by previous research (Bisello et al., 2020; Fellini, 2017). Our longer observational window highlights how this tendency seems to continue also in the post-crisis period. An increased job polarisation means that the distance between workers employed in the first and the last socio-occupational classes has widened during and after the crisis. As the likelihood of belonging to each socio-occupational class significantly varies according to the origin, these results also suggest that job polarisation may not have affected all immigrant workers in the same way and, therefore, has contributed to reinforcing inequalities between different groups of migrants.

## Conclusion

In this paper, we analyse the socio-occupational integration of migrants in Spain before, during and after the Great Recession. More specifically, we focused on the probabilities that migrants would belong to different socio-occupational classes according to their origin, controlling for their level of education, years of experience in the hosting labour market and gender. The contributions of the paper are twofold. On the one hand, compared with existing research conducted in the Spanish case, we take into account the post-crisis period. On the other hand, for the first time to our knowledge, we conduct a socio-occupational class analysis that aims to predict the probability of being located in one socio-occupational class over another, according to the origin, for each of the abovementioned periods. Thanks to this focus on the different socio-occupational classes, the conducted analysis provides a detailed and nuanced picture of the Spanish labour market before, during and, for the first time, after the Great Recession.

The results show that, although immigrants generally have a lower probability of belonging to the higher social classes compared to natives, there are multiple differences between migrants based on national origin. These differences, already existing in the pre-crisis period, seem to have been accentuated during the crisis and, even more, in the post-crisis period. During the three periods analysed, the immigrants who have suffered the most “ethnic penalties” were Africans and Eastern Europeans, while Schengen Europeans, British and North Americans are the group of migrants whose conditions are more similar, and, in certain cases, even more favourable than those of natives. Thus, despite the economic cycle considered, and notwithstanding the changes in the labour market, the ethnic penalty concerning some migrant groups seems to be stuck in a time warp. In short, on the one hand, the results of this research show that the tendency towards the destruction of middle-jobs and job polarization, already observed in the crisis period by previous research, seems to also continue during the post-crisis era in Spain. On the other hand, it also suggests that these tendencies may accentuate existing inequalities between groups of migrants with different origins.

This research is not exempt from shortcomings and hopefully future research will be able to address them. Firstly, we could not apply longitudinal techniques, such as an Age-Period-Cohort Analysis (Bar-Haim et al., 2019) that would be advisable to better explore the socio-occupational integration of migrants over time. Unfortunately, no data are currently available in Spain that can allow these kinds of techniques to be applied and our results are essentially descriptive. Secondly, the present study could not consider attrition, because LFS does not contain the information needed to take it into account. This is rather unfortunate as the crisis caused many Spaniards to emigrate to Northern Europe and caused some migrants to return home (La Fleur and Stanek 2016; Solís & Martínez Buján, 2016). Thirdly, the differences found between the different migrant groups could be connected with issues that are difficult to grasp with survey data: discrimination dynamics, job searching strategies, occupational preferences and so on (Kogan, 2004). Qualitative inquiries among different groups of migrants could definitely shed light on migrants’ occupational preferences and experiences in the Spanish job market before, during and after the crisis and delve into the reasons behind its “ethnostratification”. To explore employers’ prejudices and discrimination and investigate whether some occupational sectors may be more xenophobic than others, surveys or field experiments (e.g. Petzold, 2017a, b) could be conducted to assess to what extent the geographical origin constitutes a sorting criterion in the hiring process. Finally, even though we controlled for the highest educational level in our analyses, LFS does not contain any information on the field of study and on whether the achieved degree has been officially homologated or not. The differences found between the different migrant groups could be also related to the different fields of study or to the different yardsticks applied to the recognition of degrees from different countries, which can make it easier or more difficult to have formal educational qualifications of particular fields of study, or from particular countries over others, recognized. To better explore the importance of contextual factors, such as networks at the destination and the system of degree homologation, it could be useful to conduct a cross-national study comparing Spain with other countries. Future research may also want to look at differences by gender and conduct a socio-occupational analysis taking into account horizontal segregation, gendered migration and feminised occupations (Adams, 2010; Kofman, 2014). For this purpose, the work by Oesch (2003) about how the Goldthorpe class schema could applicable, or not, in increasingly feminised labour markets can provide inspiring insights. Our results and existing literature suggest that differences by gender may be present, but these were beyond the scope of the present paper and could not be explored here due to space limitations. Additionally, future studies might also want to look at regional differences. Finally, it would be interesting for future research to replicate the analysis presented here to contrast it with the situation during other crises, such as the current one caused by COVID-19.

## Annex

**Table 11:** Codification of each occupation*

Codes (CNO_2011)	Occupations
111	Members of the executive/legislative branches; managers of the public administration/social interests organisations
112	CEOs
121	Directors of administrative departments
122	Business/advertising/public relations/research/development directors
131	Production managers of agricultural/forestry/fishing manufacturing industries, of mining/construction/distribution industries
132	Directors of ICT services/professional services companies
141	Hosting companies´ directors/managers
142	Catering companies´ directors/managers
143	Directors/managers of wholesale/retail companies
150	Directors/managers of other service companies not classified under other headings
211	Medical doctors
212	Nursing/midwifery professionals
213	Veterinarians
214	Pharmacists
215	Other health professionals
221	University/other higher education (except vocational training) professors
222	Vocational training teachers (specific subjects)
223	Secondary education teachers (except vocational training specific subjects)
224	Primary education teachers
225	Early childhood teachers and educators
231	Special needs education teachers and technicians
232	Other teachers/teaching professionals
241	Physicists, chemists, mathematicians and similar
242	Professionals in natural sciences
243	Engineers (except agronomists, foresters, electrical/electronic/ICT engineers)
244	Electrical, electronic/telecommunications engineers
245	Architects, urban planners / geographers
246	Technical engineers (except agricultural, forestry, electrical, electronic and ICT engineers)
247	Technical engineers in electricity, electronics/telecommunications
248	Technical architects, surveyors/designers
251	Judges, magistrates, lawyers, prosecutors
259	Other legal professionals
261	Finance specialists
262	Organization/administration specialists
263	Technicians of tourist companies/activities
264	Technical/Medical sales professionals (except ICT)
265	Other sales, marketing, advertising/public relations professionals
271	Software/Multimedia analysts and designers
272	Databases/Networks specialists
281	Economists
282	Sociologists, historians, psychologists and other social science professionals
283	Priests of different religions
291	Archivists, librarians, curators, and similar
292	Writers, journalists. linguists
293	Creative/Performing artists
311	Draftsmen / technical drafters
312	Physical/chemical/environmental science and engineering technicians
313	Process control technicians
314	Natural science technicians/related auxiliary professionals
315	Professionals in maritime/aeronautical navigation
316	Quality control technicians from physical/chemical science and of engineering
320	Mining/Manufacturing/construction engineering supervisors
331	Laboratory healthcare technicians, of diagnostic tests/prosthetics
332	Other healthcare technicians
333	Alternative therapies professionals
340	Finance/Math support professionals
351	Commercial agents/representatives
352	Other commercial agents
353	Real estate brokers/other agents
361	Administrative/specialized assistants
362	Customs, tax and related agents who work in the public administration
363	Law-enforcement and security agents
371	Legal/Social services support professionals
372	Athletes, coaches, sport trainers; recreational activity monitors
373	Cultural, artistic, culinary activities support technicians and professionals
381	ICT operations/ user assistance technicians
382	Computer programmers
383	Audio-visual recording, broadcasting and telecommunications technicians
411	Accounting/Finance clerks
412	Materials registration, production and transportation support services clerks
421	Library/Archives clerks
422	Postal service clerks, coders, proof-readers and staff services clerks
430	Other administrative employees without customer service tasks
441	Information clerks/receptionists (except hotels)
442	Travel agency employees, hotel receptionists, telephone operators
443	Survey agents
444	Counter clerks and similar (except ticket clerks)
450	Administrative employees with customer service tasks not classified under other headings
500	Waiters and proprietary cooks
511	Salaried cooks
512	Salaried waiters
521	Store/warehouse section managers
522	Store/warehouse section sellers
530	Shop owners
541	Kiosks or flea markets vendors
542	Telemarketing operators
543	Gas station vendors
549	Other sellers
550	Cashiers (except bank cashiers) and ticket clerks
561	Nursing assistants
562	Pharmacy / health emergency assistant technicians and other health care workers
571	Home personal care workers (except babysitters)
572	Babysitters
581	Hairdressers; aesthetic, well-being and related treatments specialists
582	Tourists’ and travellers’ assistance service workers, tourist guides and similars
583	Building maintenance/cleaning; maintenance supervisors; janitors and butlers
584	Workers who own small accommodation
589	Other personal service workers
591	“Guardia civil” (one of the two Spanish national police force)
592	Cops
593	Firemen
594	Private security personal
599	Other protection/security service workers
611	Agriculture skilled workers (except in orchards, greenhouses, gardens and nurseries)
612	Skilled workers in orchards, greenhouses, gardens and nurseries
620	Ranching activities’ skilled workers
630	Skilled workers in agriculture and ranching mixed activities
641	Skilled workers in forestry/natural environment activities
642	Fishing and aquaculture skilled workers
643	Hunting skilled workers
711	Concrete and shuttering workers, steel fixers and similar
712	Masons, stonemasons, stone cutters and engravers
713	Carpenters (except cabinetmakers and metal structure assemblers)
719	Other workers in structural construction works
721	Plasterers and paste/mortar coating applicators
722	Plumbers
723	Painters, paperhangers and similar
724	Flooring, parquet laying workers and similar
725	Refrigeration/air conditioning mechanics/installers
729	Other finishing workers of the construction, installations (except electricians) and similar
731	Moulders, welders, sheet-metal workers, metal structure assemblers and similar
732	Blacksmiths, tool-making workers and similar
740	Mechanics/Machine adjusters
751	Construction electricians and similar
752	Other electrical equipment installers and repairers
753	Telecommunications/electronic equipment installers and repairers
761	Metal precision mechanics, potters, glassmakers and craftsmen
762	Graphic arts officers and operators
770	Food, beverage, tobacco industry workers
781	Wood workers and similar
783	Textile, clothing, leather and footwear workers
789	Gluers, divers, product testers, other workers, different craftsmen
811	Mineral extraction/exploitation workers
812	Metal treatment workers
813	Chemical, pharmaceutical, photosensitive material machines’ and plants’ workers
814	Workers in plants for wood treatment and transformation, paper manufacturing and paper/rubber and plastics products
815	Textile and leather products machine operators
816	Food/beverage/tobacco machine operators
817	Laundry and Dry-cleaning machine operators
819	Other fixed installations/machinery operators
820	Assemblers in factories
831	Locomotive drivers and similar
832	Operators of mobile forestry and agricultural machines
833	Operators of other mobile machines
834	Deck or engine sailors
841	Car/taxi and van drivers
842	Bus/tram drivers
843	Truck drivers
844	Motorcycle drivers
910	Domestic employees
921	Cleaning staff in offices, hotels and similar
922	Vehicles and windows hand cleaners
931	Kitchen helpers
932	Fast food preparers
941	Street vendors
942	Advertising deliverymen, shoe shiner and other street workers
943	Baggage handlers, deliverymen and similar
944	Waste collectors, waste sorters and similar
949	Other elementary occupations
951	Agricultural labourers
952	Cattle labourers
953	(Mixed) Agricultural and cattle labourers
954	Fishing, aquaculture, forestry and hunting labourers
960	Mining and construction labourers
970	Manufacturing industry labourers
981	Transport labourers, unloaders and similar
982	Replenishers
001	Armed forces officers and NCOs
002	Armed forces troops/sailors

List of occupations in the National Classification of Occupations (INE, 2011) disaggregated to three digits

**Table 12:** Codification of each socio-occupational class depending on the occupation*

I Higher grade university-level professionals and managers with 10 or more employees	II Lower grade university level professionals and managers with less than 10 employees	III Intermediate professions: administrative employees and professionals of support to the administrative management and other services	IV Small proprietors and self-employed workers	V Supervisors and workers in skilled technical occupations	VI Skilled and semi-skilled manual workers	VII Unskilled manual workers
211	141	331	500	312	522	542
221	142	332	530	313	550	583
241	143	340	584	314	589	834
251	150	351		320	620	844
261	212	352		521	630	910
271	222	353		581	762	960
281	224	361		713	770	970
291	231	363		719	781	921
292	232	371		721	820	922
282	246	381		722	511	931
283	247	382		723	512	932
262	248	383		725	541	941
265	263	411		731	543	942
259	264	412		732	549	943
242	225	421		740	561	944
243	272	422		751	562	949
244	293	430		752	571	951
245	311	441		753	572	952
223	315	442		761	594	953
213	316	443		782	599	981
214	333	444		783	611	982
215	362	450		789	612	
111	372	582		831	641	
112	373	591			642	
121	001	592			643	
122		593			711	
131		002			712	
132					724	
					729	
					811	
					812	
					813	
					814	
					815	
					816	
					817	
					819	
					832	
					833	
					841	
					842	
					843	

^*^Authors’ own translation of Annex 1 contained in Domingo-Salvany (2013)

## Footnotes

1 The immigrant assimilation model exclusively refers to the economic assimilation of migrants. However, it should be remarked that in the migration literature, the concept “assimilation” has been used to refer to different dimensions (e.g. assimilation of behaviour, cultural assimilation, social assimilation, etc.). For a thorough review of the concept, see de Palo et al. (2006).

2 For more information on the Spanish LSF and its methodology, consult INE, (2005). Information about its representativeness can be found in https://www.ine.es/epa02/repondera.htm [last accessed on October 1^st^ 2021].

3 Due to a change in the National Classification of Occupations in 2011, we have converted the original variables of 2006, 2008 and 2010. Thanks to this conversion, now the Spanish classification corresponds with the international classifications. For more information on the entire classification, see Annexes 1 and 2.

4 This categorisation is inspired by the one used by Ballarino and Panichella (2015). Following their rationale, we grouped people from North America and Europe, as “their average employment condition is similar to that of the native population, if not more favorable” (Ballarino and Panichella 2015: 343). Differently from the two authors, we preferred to use Schengen Area member states + UK and Ireland instead of EU members because using this latter category would exclude countries like Norway or Switzerland, for example, whose citizens are free to move and settle in Spain and who are economically, politically and culturally more similar to the other Schengen-Area members than to other non-EU members. Also, we excluded people from Oceania as, over all the years included, LFS only interviewed 18 people from Oceania (0.06% of the total sample).

5 It should be noted that, bringing together all migrants from the Caribbean, Central America and South America did not allow us to differentiate between Brazilians and other Spanish-speaking migrants.

6 It would be relevant to be able to take other aspects into account, such as the ethnicity or race of the respondents. However, several European countries, including Spain, do not produce data that asks for this type of information. In these countries, nationality is the most readily available, and most used, criterion of distinction (for a thorough discussion on the challenges of measuring ethnicity and immigrant origin in Europe, see Dirk et al., 2009).
